# Supplementary material for: Genotype Value Decomposition: Simple Methods for the Computation of Kernel Statistics
Source: Adv Genet (Hoboken). 2022 Apr 5;3(3):2100066. doi: 10.1002/ggn2.202100066 (PMC9744480; doi:10.1002/ggn2.202100066)
Supplement: Supplementary file 1 — Supporting Information [file GGN2-3-2100066-s001.pdf]

ADVANCED GENETICS
Open Access

Supporting Information

for *Advanced Genetics*, DOI 10.1002/ggn2.202100066

Genotype Value Decomposition: Simple Methods for the Computation of Kernel Statistics

*Kazuharu Misawa**

Supplementary Material 1. Computer program

```
import sys

import logging as timeLog

import numpy as np

import vcf

from Bio import SeqIO



def args2target(args):

    target = dict()

    target["filename"] = args[1]

    target["chrNumber"] = args[2]

    target["chr"] =  args[2]

    target["start"] = int(args[3])

    target["end"] = int(args[4])

    target["phenotype"] = args[5]

    return target


def withinRange(record, target):

    result = False

    if target["chr"] == record.CHROM:

        currentPos = int(record.POS)

        if (currentPos>=target["start"] ) and
(currentPos<=target["end"] ):

            result = True

    return result



def gen2code():
```

```
    code=dict()
    code["./."]=code[".|."]=0 # ref homo
    code["0/0"]=code["0|0"]=0 # ref homo
    code["0/1"]=code["1/0"]=1 # hetero
    code["0|1"]=code["1|0"]=1 # hetero
    code["1/1"]=code["1|1"]=2 # alt homo
    return code

def gen2mat(code, record):
    num = [ code[s.data.GT] for s in record.samples]
    size=len(num)
    m = [ np.zeros(size), np.zeros(size), np.zeros(size) ]
    for i in range(len(num)):
        m[num[i]][i]=1
    return m

def statisticsGRM(genotype, phenotype):
    #a = genotype[0] # ref homo
    b = genotype[1] # hetero
    c = genotype[2] # alt homo
    d = b + 2*c
    D = np.dot(d,phenotype)
    return  D*D

def statisticsIBS(genotype, phenotype):
    #a = genotype[0] # ref homo
    b = genotype[1] # hetero
    c = genotype[2] # alt homo
    B = np.dot(b,phenotype)
```

```
    C = np.dot(c,phenotype)
    return 2 * (B*B + C*C)

def sumOfStatistics(genotypeList, phenotype, method):
    #result = [ statisticsIBS(i,phenotype) for i in genotypeList ]
    result = [ statisticsGRM(i,phenotype) for i in genotypeList ]
    return sum(result)

# prepare time record
logFile = timeLog.getLogger("time")
fh =  timeLog.FileHandler('timeLog0.txt')
fh.setFormatter( timeLog.Formatter('%(asctime)s\t%(lineno)d\t%(message)s
') )
logFile.addHandler( fh )
logFile.log(30, "init" )

# initialize encode
code = gen2code()
#chromosome and range
target = args2target(sys.argv)

#input vcf
vcf_reader = vcf.Reader(open(target["filename"], 'r'))
sampleSize=len(vcf_reader.samples)
name2num = dict()
for i in range(sampleSize):
    name2num[vcf_reader.samples[i]]=i

genotypeList = list()
```

```
for record in vcf_reader:
    if withinRange(record, target):
        genotypeList.append( gen2mat(code, record) )

phenotype = np.zeros(sampleSize)
with open(target["phenotype"]) as f:
    for line in f:
        dollar= line.strip().split()
        phenotype[ name2num[ dollar[0] ] ] = float( dollar[1] )

loop = 1000*1000
logFile.log(30, "start" )
score0 =  sumOfStatistics(genotypeList, phenotype , "GRM")
count=0.0
for i in range(loop):
    tmp = np.random.permutation(phenotype)
    if ( score0 <  sumOfStatistics(genotypeList, tmp , "GRM") ):
        count+=1
    #print( sumOfStatistics(genotypeList, tmp , "GRM") )

print( score0, count/loop)
logFile.log(30, "end" )
```

Supplementary Material 2: Usage

python kernelGRM.py input.vcf chromosome start end phenotype_file.
